# The Co-Existence of Agranulocytosis and Stevens-Johnson Syndrome (SJS) in Carbamazepine Therapy: A Case Report

**DOI:** 10.7759/cureus.28917

**Published:** 2022-09-07

**Authors:** Saima Batool, Diana Voloshyna, Muhammad Usama, Muhammad Suleman, Qudsia I Sandhu, Laxman Nepal, Naglaa G Ghobriel, Jaina Mengar, Ahmed Soodod Mohammed Rasmy

**Affiliations:** 1 Internal Medicine, Hameed Latif Hospital, Lahore, PAK; 2 School of Medicine, University of Michigan, Ann Arbor , USA; 3 Neurology, Sheikh Zayed Hospital Rahim Yar Khan, Rahim Yar Khan, PAK; 4 Internal Medicine, Islamic International Medical College, Gujrat, PAK; 5 Medicine, Ghazi Khan Medical College, Dera Ghazi Khan, PAK; 6 Internal Medicine, Kathmandu Medical College and Teaching Hospital, Kathmandu, NPL; 7 Internal Medicine, University of Alexandria Egypt, Alexandria, EGY; 8 Medicine and Surgery, Government Medical College and New Civil Hospital, Surat, IND; 9 Obstetrics and Gynaecology, Egyptian Board of Obstetrics and Gynaecology, Giza, EGY

**Keywords:** rare side effect, carbamazepine, sjs, agranulocytosis, trigeminal neuralgia

## Abstract

The therapeutic significance of carbamazepine in individuals with trigeminal neuralgia, epilepsy, and bipolar disorder is well recognized. Although it has high effectiveness, it raises the patient's risk for some adverse effects. The relationship between carbamazepine usage and agranulocytosis is well-established. Agranulocytosis is characterized by an unusually low number of neutrophils. This disorder poses a grave hazard to the patient since they are more likely to get potentially lethal bacterial or fungal infections. Moreover, carbamazepine is one of the most common causes of Stevens-Johnson syndrome (SJS), a severe skin condition with a high mortality rate. In cases where agranulocytosis and Stevens-Johnson syndrome coexist, the prognosis is relatively poor. We report a rare case of a patient who developed agranulocytosis and Stevens-Johnson syndrome after taking carbamazepine. Neutrophils accounted for 2.1% of the patient's differential leukocyte count. Furthermore, Naranjo's scale found a score of 8 for Stevens-Johnson syndrome, placing it in the "probable" category, while a score of 9 for agranulocytosis indicated that it was a confirmed adverse reaction to carbamazepine.

## Introduction

The World Health Organization ranks adverse drug reactions (ADRs) as the 5th leading cause of mortality globally. ADR accounts for 5 to 8% of all hospitalizations worldwide. The majority of adverse drug reactions (ADR) (30-45%) are cutaneous, and they account for 2% of hospital stays [1]. One such class of drug associated with a hazardous profile is carbamazepine. Carbamazepine belongs to an anti-epileptic class of medicines, most prescribed to patients with mood disorders, a history of seizures, chronic pain, trigeminal neuralgia, and fibromyalgia [2]. It is a part of the model list of essential medicines by WHO and is more tolerable in controlled-release formulations than other anti-epileptic drugs. Several rare side effects of carbamazepine have been studied, and additional research is being conducted on the onset of Steven Johnson's syndrome (SJS).

Agranulocytosis has also been linked to carbamazepine use, although it is uncommon compared to other adverse effects [3]. Based on the review of the literature, the annual incidence of SJS-TEN (toxic epidermal necrolysis; hypersensitivity reaction) with carbamazepine is one in every 10,000 newly exposed individuals [4]. In contrast, the overall risk of carbamazepine-associated agranulocytosis is minimal, with roughly six patients per million people developing agranulocytosis each year [5]. No research has been conducted on the co-existence of agranulocytosis and Steven-Johnson syndrome in a patient undergoing carbamazepine therapy and its cause of occurrence. This case study explores how carbamazepine therapy may cause agranulocytosis and SJS in patients, and it emphasizes the significance of using caution when prescribing carbamazepine.

## Case presentation

**Figure 1:**
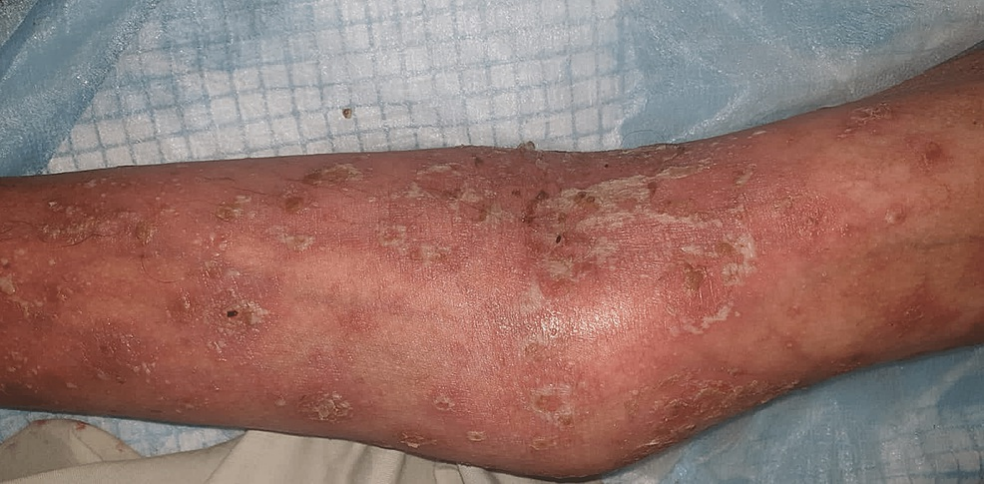
Targetoid bullous lesions on the forearm

**Figure 2:**
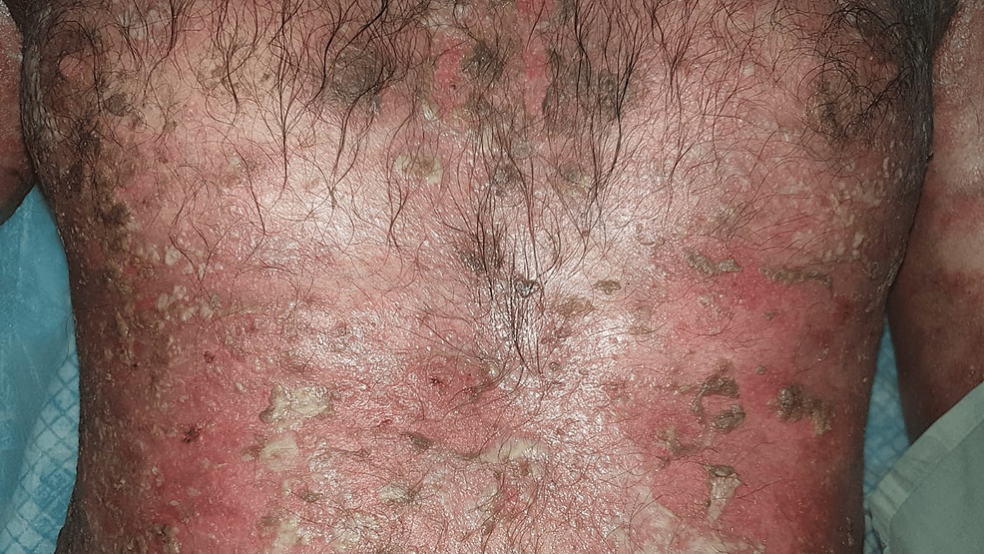
SJS involving the ventral surface of the trunk SJS: Stevens-Johnson syndrome

A 36-year-old school teacher presented to the emergency department of our tertiary care hospital with a one-week history of fever, productive cough, and the recent emergence of rashes on his arms and torso for three days. He was diagnosed with trigeminal neuralgia two weeks ago, and 200 mg of carbamazepine twice a day was initially prescribed due to the mild nature of the patient's pain. The same dose was maintained as the patient reported pain relief. However, a few days after the treatment, the patient began to feel lethargic and exhausted. Additionally, he has developed a severe cough that is accompanied by sputum and a high temperature. He self-medicated with 500 milligrams of paracetamol twice a day. Nevertheless, his health failed to improve. Furthermore, he developed erythematous rashes over the ventral surface of the torso, forearms, and axilla and fluid-filled bullae that easily ruptured. As his condition worsened, he was referred to our emergency department. On examination, the patient appeared lethargic and confused and had a temperature of 104 degrees Celsius. On his arms and trunk, erythematous, target-like lesions were observed with involvement of the buccal mucosa (Figures 1, 2).

A wide array of investigations were performed, including complete blood count (CBC), C-reactive protein (CRP), antinuclear antibodies (ANA), liver function tests (LFTs), and renal function tests (RFTs), as well as blood culture and serum electrolytes. His complete blood work revealed neutropenia, with a total neutrophil differential count of less than 2.1% (day one of admission). His total white blood cell (WBC) count was 570 cells per cubic millimeter, his lymphocyte count exceeded 77%, and his monocyte count was 21%. The patient was diagnosed with agranulocytosis after considering his clinical presentation and WBC profile. His intake of carbamazepine was stopped immediately and replaced with 100 mg of gabapentin per day. The presence of *Streptococcus pneumoniae *in the sputum culture prompted the intravenous administration of 500 milligrams of meropenem along with amoxicillin and clavulanic acid in a high dose of 1 g. Following an immediate consultation with a dermatologist and a comprehensive examination of the patient's lesion, yielding a SCORTEN (Score of Toxic Epidermal Necrosis Scale) score of 1 (Table 1), the patient was diagnosed with Steven-Jonsson syndrome (SJS). Immediately, a strong dose of hydrocortisone was administered intravenously to the patient (100 mg three times). Typically, petrolatum gauze dressings were applied on the lesions in conjunction with supportive care.

**Table 1:** SCORTEN assessment values SCORTEN score assessment provided a value of 1 for the patient. SCORTEN: Score of Toxic Epidermal Necrosis Scale; BSA: Body surface area; bpm: Beats per minute

Factor	Score = 0	Score = 1	Patient's Score
Age	≤40 yrs	>40 yrs	33 yrs
% of BSA with epidermal detachment	≤10%	>10%	15%
Heart rate (bpm)	≤120	>120	90
Presence of malignancy	No	Yes	No
Blood urea nitrogen	≤28 mg/dl	>28 mg/dl	18 mg/dl
Blood glucose (random)	≤252 mg/dl	>252 mg/dl	152 mg/dl
Serum bicarbonate	≤20 mEq/L	>20 mEq/L	18 mEq/L
Total	0	7	1

For the next five days, serial CBCs were performed daily. The patient's WBC count improved gradually, with a neutrophil percentage of 48% on the fifth day following admission (Table 2).

**Table 2:** Blood counts of the patient during the hospital stay N-neutrophils, L-lymphocytes, M-monocytes.

Day	Neutrophils	Differential leucocyte count (%)		
1	570	N – 2.31	L – 77	M – 21
2	890	N – 6.31	L – 74	M – 19.7
3	1400	N – 15.7	L – 68.0	M – 16.33
4	2700	N – 32.3	L – 57.0,	M – 11.7
5	5900	N – 46.0	L – 52.4	M – 1.6

Hence, based on the patient's presentation and a score of 9 on Naranjo's scale for agranulocytosis and 8 for SJS caused by carbamazepine, the diagnosis of carbamazepine-associated agranulocytosis and SJS is conclusive (Table 3). 

**Table 3:** Naranjo Adverse Drug Reaction Probability Scale Naranjo et al. [6] SJS: Stevens-Johnson syndrome

Questions	Yes	No	Do not know	Agranulocytosis	SJS
1. Are there previous conclusive reports on this reaction?	1	0	0	1	1
2. Did the adverse event appear after the suspected drug was administered?	2	-1	0	2	2
3. Did the adverse reaction improve when the drug was discontinued or a specific antagonist was administered?	1	0	0	1	1
4. Did the adverse event reappear when the drug was re‐administered?	2	-1	0	0	0
5. Are there alternative causes (other than the drug) that could on their own have caused the reaction?	-1	2	0	2	2
6. Did the reaction reappear when a placebo was given?	-1	1	0	1	1
7. Was the drug detected in blood (or other fluids) in concentrations known to be toxic?	1	0	0	0	0
8. Was the reaction more severe when the dose was increased or less severe when the dose was decreased?	1	0	0	1	0
9. Did the patient have a similar reaction to the same or similar drugs in any previous exposure?	1	0	0	0	0
10. Was the adverse event confirmed by any objective evidence?	1	0	0	1	1
Total score :				9	8

Following the diagnosis, the patient received treatment and supportive care in the hospital for the next five days. His fever subsided, his lesions receded, and scaling was observed, and he appeared to be well-oriented as his condition dramatically improved and he showed signs of a significant recovery. A decision to discharge the patient was made. His antibiotics (amoxicillin and clavulanic acid 1.2 g) and wound dressings were continued at home for his convenience, and a two-week follow-up after discharge was advised. At the subsequent checkup, the patient was found to be completely healthy.

## Discussion

Carbamazepine is the first-line treatment for generalized tonic-clonic seizures and the second-line treatment for focal-onset seizures. It is one of the most commonly prescribed drugs for treating focal epilepsy all over the world. It is extensively prescribed in Europe, South America, Africa, and Asia and is included in the model list of essential medicines by the World Health Organization. In terms of efficacy, carbamazepine formulations with controlled release are more tolerated [7]. A small number of side effects of carbamazepine have been documented.

Carbamazepine is associated with atrioventricular conduction abnormalities and sinus node dysfunction. In addition, it is known to increase the incidence of cardiorespiratory arrest [8,9]. Long-term usage of carbamazepine contributes to a drop in neutrophil count in patients. This condition, known as agranulocytosis, is characterized by an abnormally low white blood cell count and can result in many infections. Agranulocytosis has been observed in long-term treatment patients. The disease has a 10% mortality rate, and 80% of patients recover completely. The most common infections associated with agranulocytosis are pneumonia, tonsillitis, pharyngitis, and stomatitis [10]. People over the age of 60 years have twice as many cases of agranulocytosis as those younger than 60 years [11]. Multiple patients with schizoaffective disorders have been prescribed carbamazepine for mood stability. Twelve years ago, a 50-year-old patient with schizoaffective disorder was prescribed carbamazepine. His blood tests demonstrated a decline in the WBC count over the years, with no significant improvement. Eventually, the medication had to be discontinued to prevent further WBC decline and subsequent infections. Patients receiving carbamazepine should undergo three-monthly blood tests to monitor the complete blood count. Treatment often entails stopping exposure to the offending substance and then administering broad-spectrum antibiotics intravenously to either prevent or treat any resulting illnesses [12].

Antiepileptic medications, including carbamazepine, are regarded as a common cause of Stevens-Johnson syndrome. A study of the incidence of SJS with the use of several AEDs revealed a 9-fold increase in risk with AED use and a 20-fold increase with carbamazepine, phenytoin, lamotrigine, rufinamide, zonisamide, and clorazepate [13]. Within nine days of starting carbamazepine therapy, a 36-year-old epileptic patient of Sri Lankan descent developed extensive blisters, according to a case report [14]. Studies indicate that the (HLA)B*1502 allele is associated with carbamazepine-induced SJS in Indian Asians. Therefore, it is essential to undergo genetic testing on those who are at risk, before prescribing carbamazepine [14,15]. In a 22-year-old woman with a history of seizures, levetiracetam administered in combination with carbamazepine enhanced the incidence of SJS, characterized by vesicles and erythematous papules. The condition improved following administration of levetiracetam, and carbamazepine was discontinued [16]. SJS mediated by carbamazepine can also arise in people with no history of seizures but who complain of bipolar syndrome-like symptoms. In a similar case report, a 29-year-old woman diagnosed with bipolar disorder was administered carbamazepine during therapy, which triggered the onset of SJS within 14 days of the medication [17]. There are limited therapeutic options available for Stevens-Johnson syndrome. SJS treatment options include replenishing electrolytes, pain control, wound care, infection control, and nutritional support.

Steven-Johnsons syndrome can be treated with immunosuppressive and immunomodulating drugs such as corticosteroids, cyclosporine, intravenous immune globulin, monoclonal antibodies, and antitumor necrosis factor [18]. Cyclosporine (cyclosporin A) is a conveniently available and cost-effective alternative for the treatment of SJS, resulting in improved skin condition and re-epithelialization within one week of therapy [19]. The Stevens-Johnson syndrome and agranulocytosis are consequences of carbamazepine treatment for epilepsy or other conditions. There is only one case report in which carbamazepine produced both agranulocytosis and Stevens-Johnson syndrome in the same patient. After eight days of taking 200 mg of carbamazepine orally, the patient exhibited signs of agranulocytosis and Stevens-Johnson syndrome. The SCORTEN score of one was used to make the SJS diagnosis. The Naranjo's scale found a score of 7 for Stevens-Johnson syndrome, placing it in the "possible" category, whilst a score of 9 for agranulocytosis indicated that it was a confirmed adverse reaction to carbamazepine [4].

## Conclusions

Caution must be taken when initiating carbamazepine therapy. Although it is inexpensive, generally well-tolerated, and useful in a number of contexts, it does not come without risks to the health of patients who use it. It is crucial to monitor white blood cell counts during carbamazepine therapy, as the early detection of agranulocytosis can lead to a reversal of white blood cell counts, potentially preventing the advent of severe infectious complications. Moreover, due to SJS's rarity and potential lethality, a complete medication history from the patient is crucial. Timely intervention in at-risk patients can result in significant morbidity and mortality benefits. Therefore, appropriate risk assessment, patient education, and proper follow-up after the initiation of carbamazepine are of utmost importance.
